# Precision-based exercise as a new therapeutic option for children and adolescents with haematological malignancies

**DOI:** 10.1038/s41598-020-69393-1

**Published:** 2020-07-30

**Authors:** Francesca Lanfranconi, W. Zardo, T. Moriggi, E. Villa, G. Radaelli, S. Radaelli, F. Paoletti, E. Bottes, T. Miraglia, L. Pollastri, P. Vago, F. Nichelli, M. Jankovic, A. Biondi, A. Balduzzi

**Affiliations:** 1 Paediatric Haematology-Oncology Unit, Department of Paediatrics, University of Milano-Bicocca, MBBM Foundation/ASST Monza, Maria Letizia Verga Center-Via Cadore, 20900 Monza, Italy; 2 Cattolica University - Department of Pedagogy, Milan, Italy

**Keywords:** Physiology, Diseases, Health care, Medical research, Oncology

## Abstract

Children and adolescents with haematological malignancies (PedHM) are characterized by a severe loss of exercise ability during cancer treatment, lasting throughout their lives once healed and impacting their social inclusion prospects. The investigation of the effect of a precision-based exercise program on the connections between systems of the body in PedHM patients is the new frontier in clinical exercise physiology. This study is aimed at evaluating the effects of 11 weeks (3 times weekly) of combined training (cardiorespiratory, resistance, balance and flexibility) on the exercise intolerance in PedHM patients. Two-hundred twenty-six PedHM patients were recruited (47% F). High or medium frequency participation (HAd and MAd) was considered when a participant joined; > 65% or between 30% and < 64% of training sessions, respectively. The “up and down stairs'' test (TUDS), “6 min walking” test (6MWT), the “5 Repetition Maximum strength” leg extension and arm lateral raise test (5RM-LE and 5RM-ALR), flexibility (stand and reach), and balance (stabilometry), were performed and evaluated before and after training. The TUDS, the 5RM-LE and 5RM-ALR, and the flexibility exercises showed an increase in HAd and MAd groups (*P* < *0.05*), while the 6MWT and balance tests showed improvement only in HAd group (*P* < *0.0001*). These results support the ever-growing theory that, in the case of the treatment of PedHM, ‘exercise is medicine’ and it has the potential to increase the patient’s chances of social inclusion.

## Introduction

One high-risk group susceptible to the effects of physical inactivity are children and adolescents (Ped) who survive haematological malignancies (HM). The growing survival trends are due to joint efforts to fight HM all over the world since the seventies: effective therapies, including hematopoietic stem cell transplantation (HSCT), multicentre randomized clinical trials, biological stratification and tailored therapies based on the evaluation of treatment response (mainly by the assessment of minimal residual disease), contributed to progressively improve outcomes. In Italy, cancer mortality rates in children were three-fold higher in the early seventies than in 2008^1^. In addition, 5-year survival after cancer diagnosis increased in the last three decades, reaching 82% in children and 86% in adolescents^1^. The expected number of new cancer cases in children aged between 0 and 14 years of age is approximately 7,000 in the period 2016–2020, while the corresponding figure for adolescents between 15 and 19 years of age is 4,000 according to the Associazione Italiana di Ematologia e Oncologia Pediatrica (AIEOP)^1^. In the European Society of Paediatric Oncology’s (SIOPE) perspective, today there are nearly 500,000 European PedHM survivors and in 2030 this number will be around 750,000. Out of this two-thirds of the patients will have some late effects and/or sequelae due to treatment and/or complications. Such late effects/sequelae are severe in half of the cases and have a strong impact on the patient’s daily life^2^. SIOPE is committed to improving awareness of childhood cancer survivors in order for them to take responsibility for their own follow-up and to encourage national health systems to address the issues of long-term follow-up. As PedHM survivors move from the beginning of cancer treatment into their full potential as young adults, many of them would be well advised to embrace many of the 2030 Sustainable Development Goals, as identified in the 2016 Bangkok declaration on physical activity for global health and sustainable development^3^. A global glance at childhood cancer survivors invariably shows poor levels of fitness that are continuous and life-long, even after all cancer treatment, though there are a limited number of high-quality studies available in this area of research^4^.

Physical activity levels in PedHM patients are most definitely influenced by access to safe, affordable and proper programs and places in which to be physically active. It may be useful, therefore, to introduce tailored training programs that might be carried out directly in the cancer centres, where PedHM patients already spend a long time for their treatment as inpatients or are followed up as outpatients. Implementation of innovative action plans targeting PedHM patients should foster collaboration across centres, guided by a shared vision to realize the multiplicative benefits of a more active world, especially in the hospital settings^5^. The “Physical activity strategy for the WHO European region 2016–2025” has a specific priority in ensuring that opportunities for physical activity are included in care planning and practice and are available in long-term residential care settings^6^. It seems that patients with physical disability, such as PedHM survivors, will be willing to engage in physical activity when they have a positive attitude, perceive that there is social support, and believe in their own ability to perform at a high level^7^. Increased physical health also correlates with better emotional health and vocational identity, both affected by the indirect consequence of treatment intensity and age at diagnosis^8^.

### Reduced exercise ability in PedHM patients

Exercise capacity in PedHM patients is poorly addressed in scientific literature and long term follow up (over 3 years) of their physical performance is neglected. In comparison, we know that most adult acute myeloblastic leukemia survivors achieve full recovery of quality of life 3 years after intensive chemotherapy, not considering the fact that their exercise capacity remains limited: basic functions such as walking capacity show poor recovery gains on average and do not return to normal even 3 years after the eradication of disease^9^. Guardian and children’s/adolescents’ reports of quality of life (QoL) during chemotherapy for HM indicate that adverse effects due to drug toxicity are the primary factors affecting physical performance resulting in fatigue^10^. Among the potential consequences of childhood cancer therapy are death, cardiomyopathy^11,12^, pulmonary diseases^13–16^, myopathy^12,17,18^, osteonecrosis (ON)^19^ and motor nervous function impairment^20^. Exercise tolerance is severely reduced in PedHM patients when compared to healthy peers^18,21,22^. Strength is also reduced at the start of treatment^23,24^, after being off-therapy for 1 year^25^ and even after 15 years of follow-up^22^. One year after stopping therapy, balance is also negatively affected in PedHM patients^26,27^. Other consequences of cancer treatment are pain, fatigue, anxiety and fear, which further jeopardize the possibility to perform physical activity^28^. When compared to their peers, PedHM patients are less active and more prone to engage in sedentary behaviour^29,30^.

### Precision exercise in PedHM patients

Theoretically, precision exercise is a medicine that could improve the development of basic motor skills, as well as musculoskeletal development and the ability to achieve energy balance and weight control to counteract the acute and long-term effect of treatment and its burdening aftermath. A recent Cochrane review stated that the effects of physical exercise interventions for childhood cancer survivors are not yet convincing^31^. Nevertheless, a very recent study on a larger cohort of patients showed how supervised in-hospital exercise intervention for children with cancer is safe and plays a cardioprotective role^32^. There are positive intervention effects for body composition, flexibility, cardiorespiratory fitness, muscle strength, and health-related QoL (cancer-related items)^22,33–37^. Also, patients undergoing a HSCT can benefit from adapted training^38–40^.

Precision exercise is the new frontier in clinical exercise physiology helping to induce oxidative metabolism (i.e. the main system of energy supply within cells) and boost the adaptive response of bones in vulnerable patients. Cancer treatment is associated with fluctuations of symptoms and moods during the course of the illness that can greatly influence an individual's motivation to train. In children with cancer, some physical activity barriers included physical complaints and safety concerns that were more pronounced in on-therapy childhood cancer patients but also persisted off-therapy^41^. Collectively, these fluctuations emphasize the critical need for the precision exercise prescribed by experienced medical doctors/exercise physiologists that can be adapted in response to individual clinical conditions^42^. The use of generic exercise prescriptions may actually be masking the full therapeutic potential of exercise treatment in the oncological setting. Essentially, the manipulation of training variables such as volume, intensity, frequency and recovery is an attempt to systematically structure training through phases to optimize physiological and psychological adaptations in an athlete that is also a patient with cancer^43^. Finally, work recovery and rest are fundamental to restore the availability of nutrients and energy substrates to replace the components needed by the systems (proteins in the muscle).

A recent innovation in our training program addressed the specific issue pertaining to the impact of the physiological adaptation of bone in PedHM patients. The finding of hormones and cytokines secreted by bone and skeletal muscle during exercise, has recently added experimental evidence to the idea that a crosstalk exists between these organs^44^. Bone through the hormone osteocalcin, promotes exercise capacity and in addition osteocalcin regulates the endocrine function of skeletal muscle because it boosts the expression of interleukin-6 (IL-6). It seems that a feed-forward loop between bone and skeletal muscle is essential for exercise capacity. This endocrine regulation of exercise biology suggests adapted strategies for the inhibition of muscle loss process and bone damage, such as ON in PedHM patients. A specific stimulus is needed to impact the adaptive response of bone in healthy children, e.g. plainly the physical distortion of bone cells, rather than the metabolic or cardiovascular strains classically related with exercise^45^. Perhaps the most compelling evidence that mechanical loading is indispensable to bone integrity comes from studies of bed rest, space flight, and spinal cord injury, which showed that bone loss is rapid when mechanical forces acting on the skeleton are markedly diminished^46^. In order to reduce the risk of bone damages in PedHM patients with articular ON, we provided adapted training, but avoided the immobilization of these patients and included activities that create high ground-reaction forces, such as jumping, skipping, running and strengthening exercises.

### Aim

This study is aimed at the evaluation of the effects of 11 weeks (3 times weekly) of precision exercise training on PedHM patients from the beginning to the end of treatment, including HSCT. Specific endpoints were: (1) individual exercise tolerance; (2) skeletal muscle strength; (3) balance and flexibility; (4) QoL, i.e. the physical, social and emotional aspects that are relevant to the process of resilience and community inclusion; and (5) satisfaction with the intervention as perceived by the participants and their parents.

## Results

### Participants

**Figure 1:**
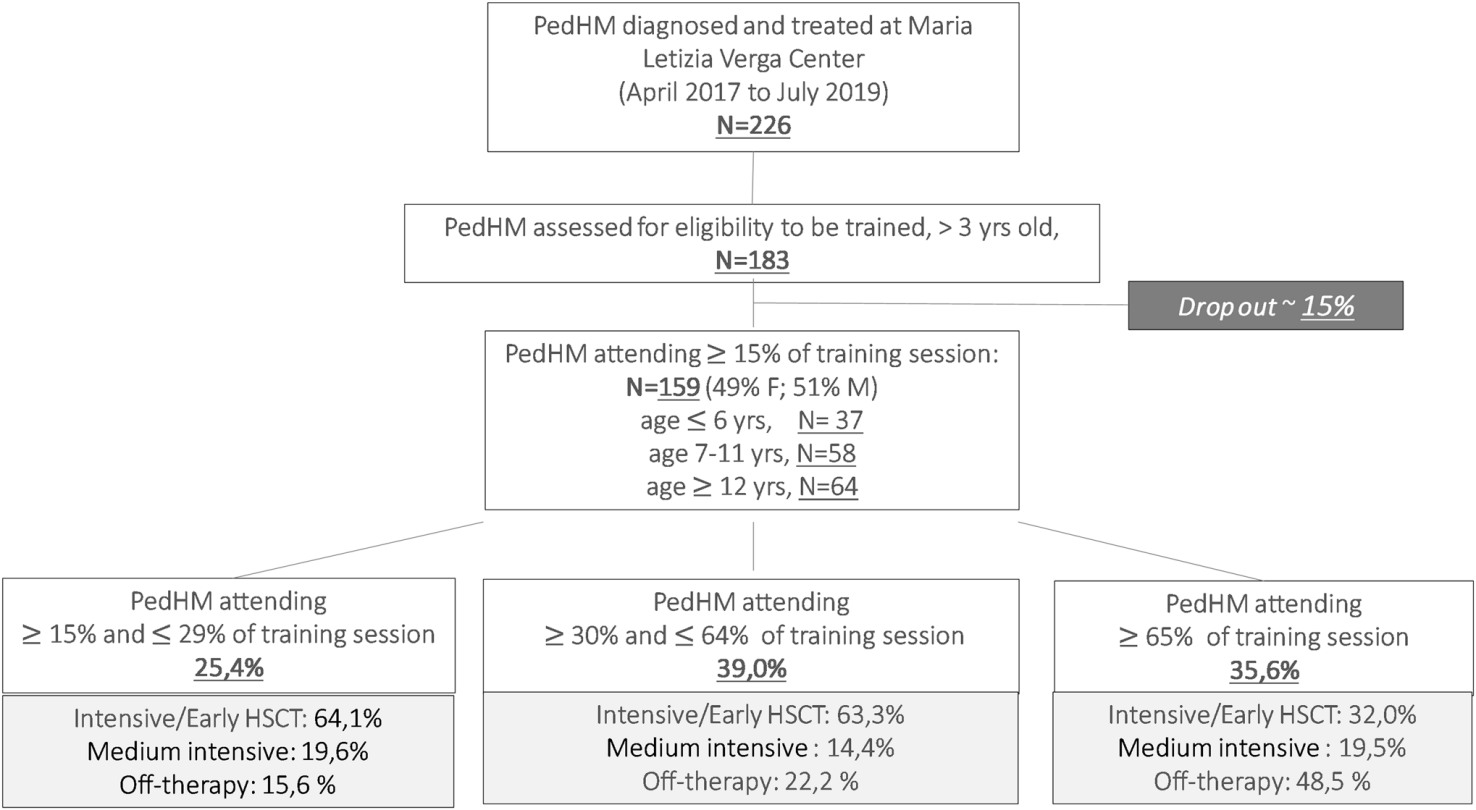
PedHM patients eligible for precision exercise training. Age, gender and clinical phases of treatment are shown. High (HAd), medium (MAd) and low (LAd) frequency adherence to training sessions are represented.

This prospective longitudinal cohort study recruited 226 PedHM patients of both genders, all older than 3 years of age who were being treated at the haemato-oncology Maria Letizia Verga centre (MLV), Monza-Italy (Fig. 1). Two male participants aged 21 years old were also admitted due to their long clinical history of functional impairment for severe ON, meaning that they were still in follow up at the center. The number of PedHM patients eligible for precision exercise training were 183 and 159 accepted the invitation to participate in a precision exercise training program. Adherence to training sessions was calculated as number of sessions (%) attended on possible 33 sessions. Figure 1 shows the adherence level to the study from April 2017 to July 2019. High frequency adherence (HAd) was indicated when a participant joined > 65% of the training sessions, between 64 and 30% was labelled medium adherence (MAd), between 29 and 15% was a low adherence (LAd). Table 1 shows the anagraphic and clinical characteristics of HAd, MAd and LAd groups. PedHM patients were affected with acute lymphoblastic leukemia (ALL), acute myeloid leukemia (AML), Hodgkin lymphoma (HL) and non-Hodgkin lymphoma (NHL). As regards to the non-oncological diseases PedHM patients were affected with aplastic anemia, drepanocytosis, adrenoleukodystrophy and thalassemia major. Patients were divided by age into three groups (3–6; 7–11; 12–18 years). Dropouts were due to logistic reasons or in cases of death (4 deceased PedHM patients). Participants were enrolled for more than 1 round when their functional capacity and exercise tolerance were far from the established goals due to an unfavorable clinical history charactherized by recurrent infections, disease relapse, severe Graft Versus Host Disease: 24 patients joined 2 or more rounds (47%). A control group of 18 healthy children (CTRL) of matching age and gender was evaluated as they participated in recreational (not competitive) activities.

**Table 1:** Anagraphic and clinical characteristics of PedHM patients divided in high (HAd), medium (MAd) or low (LAd) frequency adherence to training sessions.

	HAd	MAd	LAd
Patients (num)	57	62	40
Age (years)	11.62 ± 4.34	9.89 ± 4.46	10.62 ± 4.62
Age (years, range)	4–22	3–22	3–22
Sex (% female)	44.44	71.19	45.61
Aged 3 < x < 6 years (%)	13.65	27.13	20.00
Aged 7 < x < 11 years (%)	34.60	37.30	38.30
Aged 12 < x < 22 years (%)	53.10	35.60	41.70
Aged 12 < x < 22 years, % Female	44.20	38.10	44.00
ALL (%)	51.85	67.80	59.65
HSCT (%)	11.11	23.73	24.56
**Treatment protocol**	*AIEOP BFM ALL 2009 and 2017*		
AML (%)	51.85	11.86	10.53
HSCT (%)	4.94	8.47	7.02
**Treatment protocol**	*AIEOP AML 2013*		
HL (%)	17.28	5.08	8.77
HSCT (%)	2.47	1.69	5.26
**Treatment protocol**	*EuroNet PHL C2*		
NHL (%)	6.17	3.39	5.26
HSCT (%)	1.23	1.69	5.26
**Treatment protocol**	*AIEOP LNH97*		
Non onco/HSCT (%/%)	14.81	11.86	15.79
HSCT (%)	7.41	10.17	8.77
Deceased (num)	1	2	1

*ALL*, acute lymphoblastic leukemia; *AML*, acute myeloid leukemia; *HL*, Hodgkin lymphoma; *NHL*, nonHodgkin lymphoma; *HSCT*, hematopoietic stem cell transplant.

Each participant was informed by the referring paediatric haematologist about the possibility to participate in the research protocol. Subsequently, a sports medicine medical doctor performed an illustrative interview and an examination, including an anthropometric evaluation, baseline ECG and a respiratory test (spirometry), to evaluate the possible risks when performing precision training.

### Functional capacity and endurance

**Figure 2:**
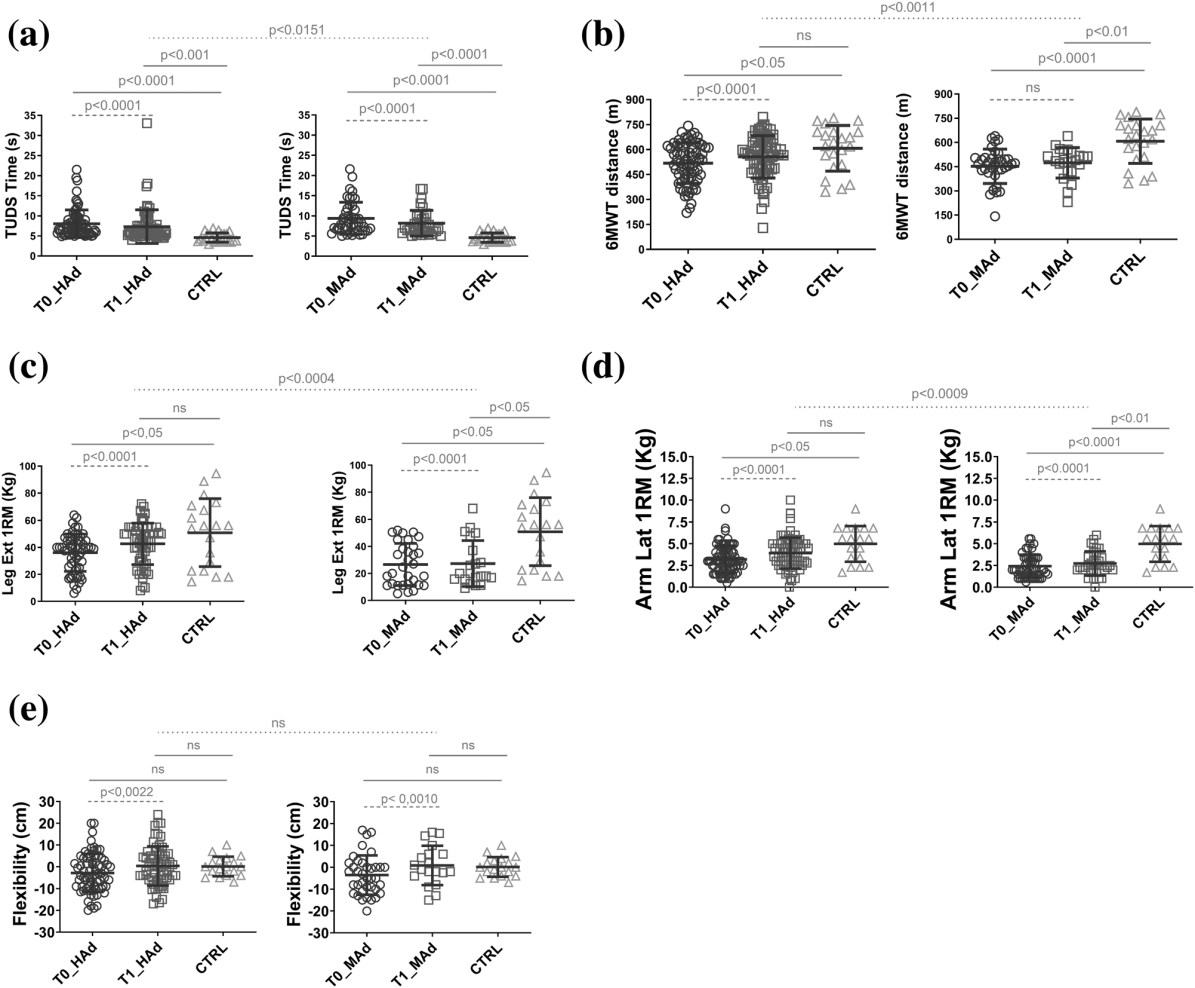
Performances during different tests by the PedHM patients before (T0, circles) and after (T1, squares) 11 weeks of precision training. The PedHM patients has been compared to the CTRL group (triangles). Left side of the panel shows the PedHM patients at high frequency adherence to training sessions (HAd), while at right the medium frequency adherence (MAd). (**a**) Timed up and down stairs test (TUDS). (b) 6 min walking test (6MWT). (**c**) strength of quadricep muscles during a leg extension test. (**d**) strength of deltoids muscles during a lateral arm raise test. (**e**) flexibility of posterior muscular chains during a stand and reach test. Continuous lines of statistical significance: ordinary one way ANOVA. Dashed lines: paired t test. Speckled line: unpaired t test.

Figure 2 panel (a) shows the performance (time) during timed up and down stairs test (TUDS) and there is a statistically significant difference after training: in HAd and MAd groups the performance improved by 13% and 18%, respectively. When compared to CTRL the HAd and MAd groups at T0 and T1 showed a lower exercise capacity: the HAd and MAd at T0 were 43% and 52% less performant than CTRL, respectively; the HAd and MAd at T1 were 38% and 44%, respectively, less performant than CTRL. In the case of the HAd group, at T1 the lower exercise capacity is less marked than the MAd when compared to CTRL (p < 0.001 vs p < 0.0001). There is a statistical significance in the HAd_T1 values compared to MAd_T1, that HAd shows better performance after the training. Because the LAd group did not show any statistical difference at T0 and T1, the data is not presented in these results nor in the following ones.

Figure 2 panel (b) shows the performance (distance) during the 6-min walking test (6MWT). There was a statistically significant difference before and after training only in the HAd group; it improved by 7%. The MAd group did not show any performance improvement. When compared to CTRL, both HAd and MAd at T0 showed a lower performance capacity (15% and 26%, respectively). At T1, no difference in performance in group HAd when compared to CTRL was noticed while for the MAd group there is a statistically lower performance when compared to CTRL (21%).

### Resistance

Figure 2 panel (c) shows the strength of quadricep muscles (1RM) during a leg extension test. There was a statistically significant difference before and after training for both HAd and MAd groups that improved by 14% and 18%, respectively. Compared to CTRL, HAd were 39% less strong at T0 but no differences were noticed at T1; while MAd at T0 showed a 48% lower performance capacity and at T1 46%. There is a statistical significance in the HAd_T1 values compared to MAd_T1, that HAd shows better performance after the training.

Figure 2 panel (d) shows the strength of deltoid muscles (1RM) during a lateral arm raise test and there is a statistically significant difference after training: in HAd and MAd groups where the performance improved by 15% and 17%, respectively. Compared to CTRL the HAd and MAd groups at T0 and T1 showed a lower exercise capacity: at T0, the HAd and MAd were 35% and 52% less performant than CTRL, respectively. At T1 only the MAd were 46% less strong then CTRL. There is a statistical significance in the HAd_T1 values compared to MAd_T1, showing that HAd performed better after the training.

### Flexibility and balance

Figure 2 panel (e) shows the flexibility of posterior muscular chains during a stand and reach test and there is a statically significant difference after training: in HAd and MAd groups and the performance improved by 8% and 29%, respectively. Compared to CTRL, the HAd and MAd groups at T0 and T1 did not show any difference. No statistical significance in the HAd_T1 values compared to MAd_T1, showing that the HAd performed better after the training.

The results of balance performance using a stabilometry test are shown in Table 2 and a statistically significant difference was seen at T1 for HAd, but not for MAd. It was impossible to use the stabilometric platform in CTRL because they could not have access to the centre for safety (immunological) reasons.

**Table 2:** Balance performance at a stabilometry test before (T0) and after (T1) 11 weeks of precision training, linear regressions of performances during different tests vs PedHM patients ages and quality of life (QoL) questionnaires results.

Stabilometry	T0_HAd	T1_HAd	T0_MAd	T1_MAd
**EC**				
Average	447.2	459.6	443.8	388.4
*St dev*	*211.5*	*404.8*	*197.1*	*146.8*
*P value*	*0.9878*		0.6387	
**EO**				
Average	337.2	265.1	328.1	281.3
*St dev*	*220.4*	*104.1*	*145.2*	*111.3*
*P value*	*0.05*		0.6257	
**EO vs EC**				
*P value*	*< 0.0001*		< 0.0001	

Linear regressions	T0	T1	CTRL	Are lines different?
**6MWT**				
Equation	Y = 13.45 × X + 339.0	Y = 10.68 × X + 411.1	Y = 35.64 × X + 266.9	Yes
*P value*	< *0.0001*	*0.0007*	< *0.0001*	
R square	0.2259	0.1435	0.6474	
Cut off values (m)				
3–6 years: 261.98; 7–11 years: 386.84; > 12 years: 426.64				
**TUDS**				
Equation	Y = − 0.3482 × X + 12.68	Y = − 0.2951 × X + 10.53	Y = − 0.3005 × X + 8.558	No
*P value*	< *0.0001*	*0.0007*	< *0.0001*	
R square	0.1642	0.252	0.5542	
Cut off values (s)				
3–6 years:16.32; 7–11 years: 12.87; > 12 years: 10.56				
**Leg extension**				
Equation	Y = 2.658 × X − 2.317	Y = 2.597 × X + 5.228	Y = 4.677 × X − 5.408	No
*P value*	< *0.0001*	< *0.0001*	< *0.0001*	
R square	0.417	0.3345	0.6677	
Cut off values (kg)				
3–6 years: 3.83; 7–11 years: 9.43; > 12 years: 21.62				
**Lateral raise**				
Equation	Y = 0.2644 × X − 0.3149	Y = 0.2640 × X + 0.2334	Y = 0.3486 × X + 0.7754	No
*P value*	< *0.0001*	< *0.0001*	< *0.0001*	
R square	0.47	0.42	0.61	
Cut off values (kg)				
3–6 years: 0.75; 7–11 years: 1.09; > 12 years: 1.94				
**Flexibility**				
Equation	Y = 0.0013 × X − 4.46	Y = 0.3149 × X − 3.643	Y = − 0.0173 × X + 1.326	No
*P value*	*0.9944*	*0.2231*	*0.521*	
R square	< 0.0100	0.02	0.03	
Cut off values (cm)				
All ages: − 13.40				
**Balance EO**				
Equation	Y = − 18.05 × X + 535.6	Y = − 10.31 × X + 401.2	np	No
*P value*	< *0.0001*	*0.005*	*np*	
R square	0.24	0.13	np	
Cut off values (mm)				
3–6 years: 716.85; 7–11 years: 427.85; > 12 years: 394.10				

QoL	T0_TOT	T1_TOT	T0_Phys	T1_Phys
**Children**				
Average	71.77	74.31	66.51	76.47
*St dev*	*3.02*	*5.83*	*9.75*	*3.39*
*P value*	*0.0021*		0.0068	
**Parents**				
Average	67.94	68.42	62.46	71.12
*St dev*	*5.56*	*7.01*	*10.94*	*4.39*
*P value*	*0.0019*		0.0099	

*HAd and MAd*, high and medium frequency adherence of training sessions; *EC*, eyes closed; *EO*, eyes open; *TUDS*, timed up and down stairs test; *6MWT*, 6 min walking test.

### Very frail PedHM patients characteristics

**Figure 3:**
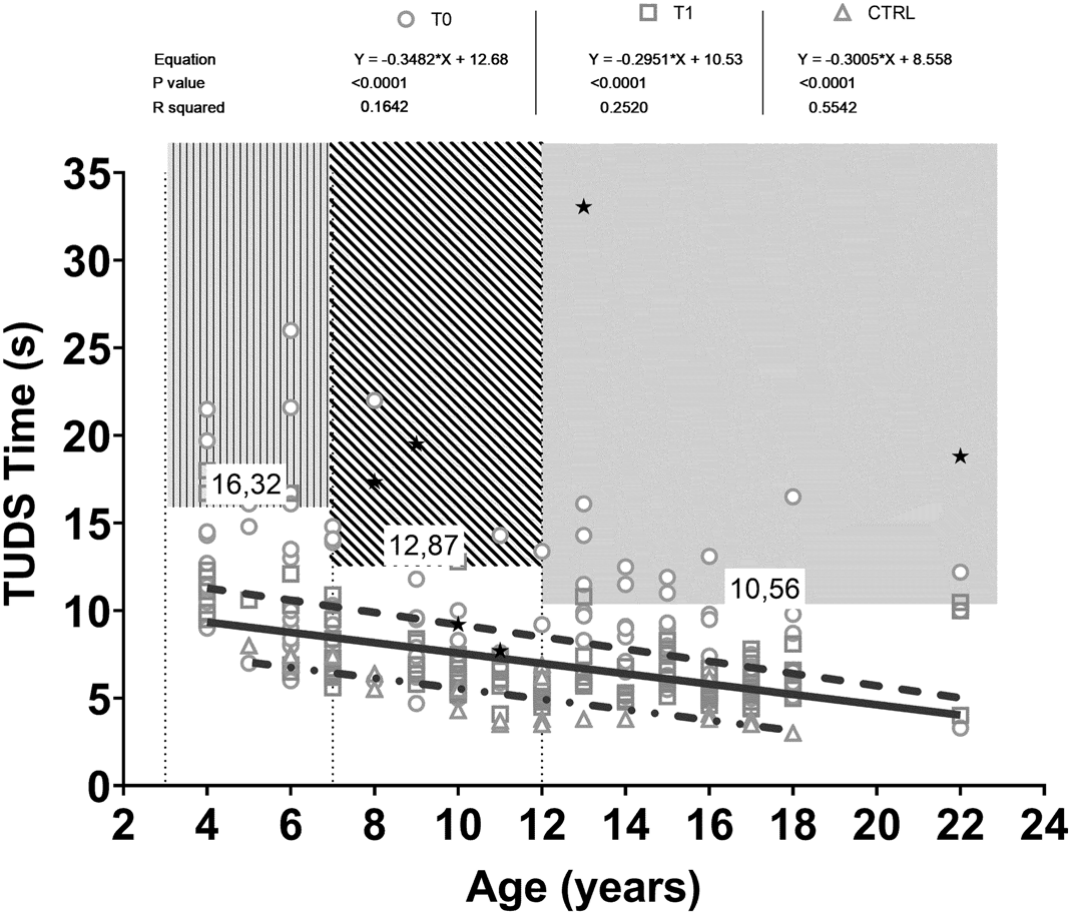
Correlation between age and performance during timed up and down stairs test (TUDS). Three age’s cohorts (3–7 years; 8–11 years; > 12 years) are represented before (empty circle and dotted line) and after (empty squares and continuous line) training and compared to CTRL (empty triangle and dotted-continuous line). PedHM patients that could not take part in the T0 evaluation session due to walking inability, are represented only after training (T1, full stars).

Figure 3 shows a negative correlation between age and performance during TUDS. The 3 age cohorts are represented before and after training and compared to CTRL. PedHM patients that could not take part in the T0 evaluation session due to walking inability are represented only after training (T1, full stars). The CTRL group showed a higher correlation (r^2^ 55.4) when compared to PedHM patients  at T0 (r^2^ 0.16) and T1 (r^2^ 0.25). A cut off value for each cohort of age has been calculated as mean + 1SD toward the worst performance at T0 and above this value fall the worst exercise capacities shown by the very frail PedHM patients The same correlation was made for all the variables represented in Fig. 2 and the regressions and cut off points are presented in Table 2.

### QoL in children and parent perception, satisfaction

There was a statistical difference reported in QoL before and after training, for all groups, both by parents and children (Table 2).

Overall, the average VAS score for the 7 rounds that were investigated during the Sport Therapy study showed considerable satisfaction by both PedHM patients and their parents (9.30 ± 1.25 and 9.64 ± 0.60, respectively).

## Discussion

An extensive number of PedHM patients have been included in this study, with varying degrees of frailty and our results conclusively support the idea that moderate to high-intensity exercise is not detrimental to PedHM patient health. The precision exercise program impacted both aerobic, strength, balance and flexibility characteristics especially in those PedHM patients with HAd attending the training sessions. In line with what has been reported by previous studies of San Juan (2007), Chamorro-Viňa (2010), Perondi (2012), Kabak (2016), Fiuza-Luces (2017), Morales (2019) the precision exercise is safe and sustainable for PedHM patients including HSCT recipients^32,35–38,47^.

The PedHM patients participated with enthusiasm in the precision exercise training: an overall high satisfaction was seen, both from participants and parents points of view. This is a unique type of intervention, in a care system with a complex clinical organization that includes taking care of the full population of the centre (around 100 new PedHM patients diagnosed yearly). The expected result of having satisfied families was an ambition, but it was not a guarantee. This is thanks in part due to the fact that frail patients are often overprotected by the clinical system and their parents, so the risks associated with training programs (risk of fall, muscle injuries, skin abrasions, community infections) can be an added stress factor into the complicated management of the PedHM patients. Thus, the result of VAS is a confirmation that the approach used to motivate PedHM patients and families, and the strategies planned by paediatricians with the sports medicine MDs were fruitful and were received with an appropriate level of acceptance. The PedHM patients and their caregivers found the training sessions to be a pleasant excuse to go out from their homes and meet with the researchers involved and other participants facing the same physical challenges. Talking about their expectation to keep a certain degree of exercise capacity even as it was constantly declining from the onset of disease, had a beneficial effect on individual level of anxiety about the exercises they can perform at home or in their rooms.

Certainly, a precision exercise training is challenging when considering the feasibility from the medical personnel point of view because physicians skilful in administering adapted and tailored exercises must be added to the personnel. The background of these personnel ranges from strong theoretical human physiology knowledge to clinical exercise pragmatic approach. The Sports medicine physician is a figure peculiar of the Italian health systems, based on preventive medicine, and this profession is evolving as a field which trains personnel overlooking exercise in chronic patients including patients with cancer. Our center’s choice was to devote economical resources in introducing both Sports medicine physicians and personal trainers in order to administer the correct amount of exercise according to the clinical conditions of the PedHM patient. Considering that clinical conditions of frail PedHM patients changes almost daily, a strong investment was made in a day-to-day consultation sessions between the oncologists and the sport therapy team. That way the prise en charge was safe, effective and in the exact range of competences for each figure involved.

Observation of the dropouts and the LAd group, showed that in certain phases of the clinical history of PedHM patients, precision exercise training is not suitable (acute infections, asthenia) and there are parents that are not very willing to consider the intervention as a therapy and/or are too apprehensive when the child shows signs of fatigue. We hope that once multi-centered studies show positive results of precision training on exercise capacity, both short and long-term, the number of dropouts will fall. PedHM with AML and HL generally afforded higher adherence of precision training, although ALL patients usually had medium and lower adherences to training. A fair percentage of HAd were represented by the frailest PedHM patients but they could partake in > 65% of the total training session: this was due to a few factors such as that they were very motivated patients, they had supportive parents, and many had a background in sport competition at regional/national level before being diagnosed. We can effectively see that all the phases of treatment allowed a high adherence to the training even if frail. PedHM patients in intensive course of treatment or at 1 year of HSCE could afford to participate mainly in training between 30 and 64% of adherence.

The TUDS evaluates specific capacities of our systems, i.e. the strength of inferior limbs during up (positive work or concentric contraction) and down (negative work or eccentric contractions). All these capacities deeply impact explosive exercise (i.e. anaerobic alactacide performance). The precision exercise greatly impacts these essential functions that allow a better QoL and the ability to sustain daily living activities. Compared to Zaino et al.^48^, our cohort of patients showed an average result of the TUDS test lower than that of children with functional deficit due to cerebral palsy. However, the frailest of our PedHM patients had a similar or worse performance. Our results correlate with those of San Juan (2007), Tanir (2012), Kabak (2016) considering the different age groups and the phases of treatment^35,47,49^. The TUDS test evaluated specific characteristics of the strength of lower limbs during uphill and downhill exercises especially when it is necessary to perform explosive exercises (via metabolic anaerobic alactacide). Also, the execution of the TUDS test requires an adequate ROM of the lower limbs, coordination for fast movements, and anticipatory and reactive posture control. The training positively impacted these functions which allow patients to thrive in everyday life. The cut-off values shown in Table 2 are useful to objectively determine the fragility of PedHM patients during TUDS.

When looking at the values present in the aforementioned areas like fragility or when a clinical history was taken, these patients have had important medical complications that compromised their tolerance to physical activity (GVHS, ON, balance disruption, fear of heights, etc.). Traditionally, PedHM patients showed performance value outside of the range of the performance of the CTRL group of the same age, especially at T0. At T1 the values get closer to those of the healthy children and adolescents, but the question that remains is: how much time do the sick children need to reach a level that is considered normal? To understand this, it is necessary to think of a long term (5–10 years) follow-up of this same population. And this follow-up is currently not available. The starting point in HAd, at T0, shows an averaged better performance when compared to the MAd probably due to the fact that in MAd there is a higher percentage of intensive phase of treatment, including early HSCT. Thus, the higher increase of performance in MAd could be influenced by the starting point, i.e. the prevailing clinical condition at the time. The impact of the precision exercise program, thoroughly tailored to individual characteristics, could be noticeable especially in frail PedHM patients because they can range from severe disability as non-ambulatory to walking, after 11 weeks of training although with a medium adherence. Generally the HAd showed better performance in all the testes at T1 when compared to T0: the lower number of early HSCT and intensive treatment, plus a higher number of teens patients, could be the reason for these discrepancies.

As seen in Table 1, not statistically different ages were found in the 3 groups, but we had more teens patients in the HAd group so a stronger performance can be characteristic of post-puberty participants. Gender was also not the same in each of the 3 adherence groups: the MAd group had a higher percentage of females than the other 2 groups. But when considering just the post puberty cohort of age (> 12 years) the differences between groups decreased, so gender does not impact the differences between groups.

Figure 2 panel B shows the positive impact of precision exercise on aerobic capacity when high adherence to training is maintained. The exercise program is made up of 60 min/week specifically devoted to aerobic training. Based on our data, it seems that lower levels of aerobic training do not impact performance, specifically the oxidative pathway in PedHM patients. Once the aerobic training reaches the target of improving the efficiency of the transport and utilization chain of O_2,_ a less pronounced difference towards CTRL is noticed. On the contrary, a MAd training does not impact the oxidative metabolism of cardio-pulmonary and skeletal muscle systems. As noticed by Geiger et al.^50^, we noticed that in sick children the 6MWT almost completely represents a maximal exercise test and it could help to grade their level of impairment safely.

Figure 3 panel A and Table 2 are useful in showing cut off values that one can use to determine the fragility of PedHM patients. When the clinical history of these patients was evaluated, they experienced various clinical complications (severe joint’s GvHD, ON, balance disorders, basophobia with vertigo, etc.). Generally, CTRL, especially teens, performed better than PedHM patients. The impact of drug treatment on health is well known while fewer studies relate to the impairment of respiratory function. But both are certainly related to the capacity of aerobic performance; walking, running and cycling. Thus, endurance training would increase, whereas profound deconditioning would decrease the peak capacity of O_2_ extraction by skeletal muscles^51^. It is possible that drug treatment ultimately impacts aerobic performance, rather than the explosive force necessary for exercises as jumping.

### Strength

Muscular strength is one of the most studied parameters in sport science literature and in PedHM patients it is mostly affected by the myotoxic effects of drugs and muscle disuse. Some studies show that after a period of training there were significant increases in strength in children with solid tumours^37^, in children with LLA 1 year after diagnosis^48^ and in children in maintenance phase of treatment^36^. The PedHM pateints have significantly less strength in their upper and lower limbs in comparison to healthy peers that are of the same age. Studies have shown that the force of a knee extension is reduced in PedHM patients during treatment, especially in the beginning stages, when compared to the equivalent CTRL group. After the 11 weeks of precision training, the difference between PedHM patients and CTRL was reduced in a non-uniform way. The results of the children aged 3–6 years become much closer to those of the CTRL group of the same age at T1. The values of all age groups were better after training, despite remaining far from the results of the CTRL group. It is possible that atrophy of the muscles is more prevalent in older patients and that strength training in the volume and intensity proposed by the precision exercise training is not enough to bring their levels of strength back to what they were before being diagnosed. To understand how much time is necessary for total recovery, a long term follow up is necessary.

The strength of the lower limbs was evaluated with the leg extension test, a method also used by Perondi et al.^36^ on children with LLA in maintenance. Our project has also allowed us to demonstrate the feasibility and validity of this test on patients who underwent a HSCT (except for the most frail with ON for the lower limbs, for precautionary purposes). After the 11 weeks of training we found a marked improvement in the PedHM group. We can also make the same considerations for the strength of the upper limbs as explained in the previous paragraph. This improvement can be attributed to the exercises performed during training to stimulate the neuro-muscular adaptations of strength of the lower limbs and to the training of the muscle groups necessary for the correct execution of the test. The group of healthy CTRL perform at a much higher level than the PedHM patients, a result that demonstrates the impact of muscular atrophy in the paediatric haematology oncology population.

On the whole, the PedHM patients demonstrate results that are on average lower compared to their healthy counterparts, both before and after precision training. As observed by the Leg Extension test, the PedHM patients values are far removed from the CTRL based on age.

The precision training strengthened the upper limbs in PedHM patients that trained and stuck to the program with HAd or MAd frequency. The strength of the deltoid muscle is positively influenced by precision training. The level of strength increases with age both in PedHM and the CTRL.

This is the first study that utilizes stabilometry to evaluate the static balance of PedHM patients. The difference between test results carried out at EO and at EC demonstrate the sensibility of the method in revealing variations of postural control. On average, the performance of PedHM HAd and MAd improves after 11 weeks of training. This improvement is more evident in PedHM HAd, yet another example of how precision training has a greater impact when it is done on a consistent, frequent basis. The results of the HAd group tended towards (p = 0.052), while the results of the MAd group were not significant. In terms of this variable, there is less of an effect of precision training. One hypothesis is that the impact of treatments on the neuromuscular system is not easily modifiable using the balance trading protocol. Adolescents are attributed with the best postural control of all the PedHM patients, both before and after training.

The stand and reach test revealed itself to be useful in determining the flexibility of PedHM patients, as it is doable by this very fragile population of patients^27^. Flexibility in PedHM patients is often compromised due to illness and treatment of the illness. Wilson et al.^52^ analysed 365 children with LLA with levels of flexibility much lower than the healthy control group. Additionally, they demonstrated that the reduced flexibility was associated with an increased risk of limited functionality. Observing the correlation between the age and flexibility of the CTRL and PedHM shows interesting results. Flexibility is independent from the age of the children and teens. On the contrary, flexibility improves after 11 weeks of training and that improvement is clearly evident in the teens patients, who improved enough to surpass the performance of the CTRL group of the same age.

The QoL of PedHM is a variable investigated by many authors and researchers. Deisenroth et al. (2016), Ness et al. (2015) and San Juan et al. (2008) showed how it was significantly inferior to the QoL of CTRL in every single area, with the largest standard deviation in the area of physical well-being^21,23,24^. Other authors San Juan et al. (2007), Marchese et al. (2004), Tanir et al. (2012) and Fiuza-Luces (2017) did not find significant improvements in the QoL of their patients after training^34,35,37,49^. In our study all of the PedHM patients showed improvements in their general QoL and particularly in the macro area of physical well-being. This result reflects their parent’s opinion as well. These results help us say, with confidence, that the experience of group training in the case of the PedHM patients provided them with a support net and close relationship that guaranteed increased motivation in therapy participation.

### Conclusion

Precision based exercise training is a challenge: it can be successful when multidisciplinary collaboration is exercised between paediatricians and sports medicine experts. A precision-based exercise program is a useful vast opportunity to improve the performance and the QoL of PedHM patients and should be considered as part of their care. We contend that PedHM patients, if supported by tailored training programs right from the diagnosis of cancer, can potentially become performant as high-level athletes. All PedHM patients can be included in precision exercise training when their age permit to sustain exercise (> 3 years) regardless of gender and the clinical history of fragility. After 11 weeks of training there is a positive impact on the chain of transport and use of O_2_, strength, balance and flexibility. Patients that adhere to more than 64% of training sessions have a greater rate of improvement than those who only take part between 30 and 64%. The latter are mostly very frail PedHM patients. No advantages were recorded in PedHM patients that observed < 15% of adherence to training sessions. PedHM patients are overall less performant than healthy CTRL of the same age and gender. A cut off value has been discovered for each test used, in order to highlight the frailest PedHM patients that need extra precautions when exposed to training protocols. In HAd group, an amelioration of QoL was noticed, mainly due to the increased exercise tolerance that impacted their social behaviour. The satisfaction of the intervention that the Sport Therapy program has provided has been extremely high.

## Methods

**Figure 4:**
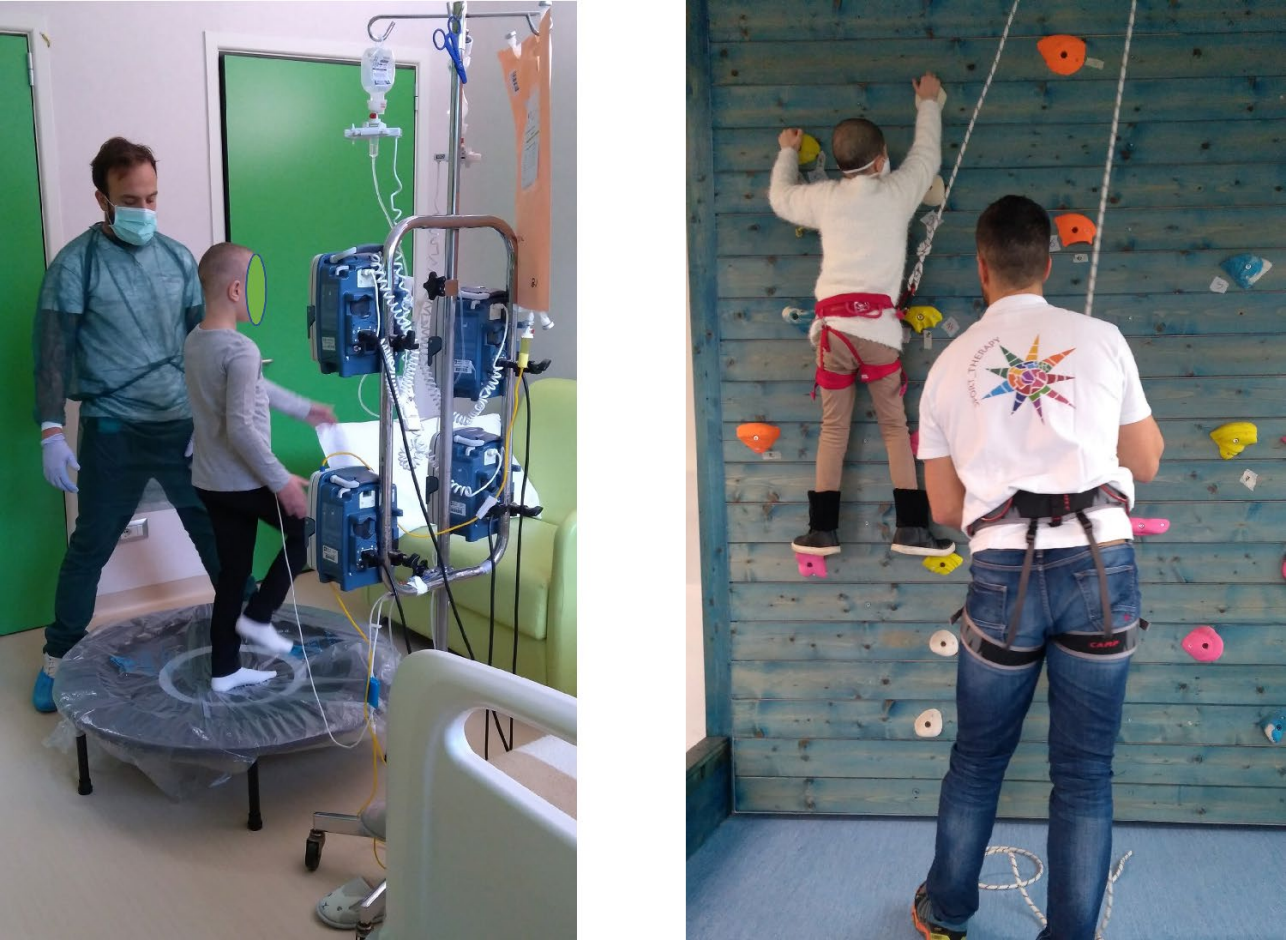
Precision exercise training made by the same patient during 2 different times of her clinical history: 2 days (in room) and 3 months (in gym, climbing wall) after haematological stem cells transplant.

Every attempt to avoid any unnecessary discomfort and disturbance to all PedHM patients and parents was made according to the ERICE statement about the cure and care of long-term survivors of childhood cancer^53^ and according to the Declaration of Helsinki as a statement of ethical principles for medical research involving human participants. All children and both parents provided informed assent and written consent to participate in the project, respectively, which was approved by the University of Milano Bicocca ethics committee (registered number 2017/284). As regard Fig. 4, both child and her/his parents provided an informed consent for an online open-access publication. Personal data were treated according to the European standard principles of confidentiality (n. 2016/679).

### Precision exercise protocols

PedHM patients were divided in 2 precision exercise protocols depending on their clinical history and according to the intensity of their chemotherapy protocol. A “frail training protocol” was used for intensive lines of treatment: (1) patients in their early post-transplantation course (< 9 months); (2) patients affected with ALL and AML, during their front-line induction. A “standard training protocol” was used for medium intensive lines of treatment: (1) patients affected with ALL during their consolidation, high-dose blocks, and re-induction, or after relapse; (2) patients with AML, after the induction phase; (3) patients in late post HSCT course; (4) patients affected with NHL and HL. PedHM patients off therapy were considered in their early follow up course (< 6 months). Besides the stratification according to the underlying disease, each patient was evaluated for his/her performance status at the onset and was continuously reassessed during each treatment, so that the risk stratification and the consequent training protocols was a dynamic process. Indeed, chemotherapy protocols, mainly those for ALL treatment, and the post-transplant course include high cumulative steroid dose with severe repercussions on PedHM patient performance due to fatigue, nausea, myalgia, etc. Moreover, the vast majority of PedHM have low haemoglobin levels, despite frequent packed red blood cell transfusions. Toxic and infectious complications may affect every organ and system. Transplanted patients often suffer from lung infections, requiring O_2_ support. PedHM patients during their long-term follow-up, especially ALL and transplanted patients, are affected by avascular ON.

PedHM patients were trained either directly in-patient rooms in one of the 3 wards (Bone Marrow Transplant Center, Haematology and Day Hospital) or in the gym at MLV Centre (Fig. 4). Depending on the clinical condition of each PedHM patient, 2 different training modules were performed: each session, of 20–35 min (frail patients) or 50–60 min (standard patients) duration, was supervised by a medical doctor with a specialization in Sports Medicine and 1 sport scientist, with a ratio between patient/therapist of 1:2. Safety parameters such as heart rate (HR) and O_2_ saturation, as well as training intensity and adherence to training sessions were documented daily in the patient’s record.

### Evaluation of the QoL and satisfaction

A child self-report (divided per age’s cohorts) and a parent proxy-report inventory (PedsQL 4.0) were used to measure the participants’ QoL^54^. The PedsQL 4.0 is a modular approach to measuring health-related quality of life in healthy children and adolescents and those with acute and chronic health conditions. There are 3 Summary Scores: total, physical health and psychosocial health summaries. Each item has a score between 0 and 4, so the scale score is between 0 and 100. Higher scores indicate a better QoL.

The visual analog scale (VAS) method was used to evaluate the satisfaction of both participants and their parents after the precision training intervention. The visual analog scale (VAS) is a patient-reported scale from 0 to 10 (where 0 is not satisfied and 10 is completely satisfied) that is increasingly being used to assess outcomes such as patient satisfaction after different medical interventions^55^.

### Exercise tolerance capacity

The TUDS was performed accordingly to the Zaino’s et al. protocol^48^ in order to measure the general neuro-muscular aspect on the control of posture: it was, in fact, hypothesized that to carry out the test the participant must possess a certain strength of the lower limbs and the abdominal muscles, as well as have a good coordination in the upper limbs and lower limbs movement, to go up and down the stairs as fast as possible.

The 6MWT was performed according to the Geiger’s et al. protocol^50^ with a meter-counting wheel (Silverline 868793 Mini measuring wheel). During the test SpO_2_ and heart rate (HR) were continuously monitored by the Pulse saturimeter (Oxy True A-Blue Point Medical, Selmsdorf, Germany)^50^.

The strength of the limbs has been investigated through the execution of the test of the 5 maximum repetitions (5RM), which evaluates the maximum strength exerted by the participant before reaching muscular exhaustion according to Fiuza-Luces’ et al. protocol^37^. For the lower limbs, the force of the extensor muscles of the knee was evaluated by using adaptable machinery that varied according to the size of the patient’s developmental age (Leg extension Alpha pro, multi-function bench, Kettler, Ense-Parsit, Germany). For the upper limbs (deltoid, trapezius and supraspinatus) the force test was carried out through the lateral arm raises with dumbbells (Toorx, Pozzolo Formigaro, Italy).

A stabilometric board was used in order to evaluate the balance of participants (TecnoBody, Dalmine (BG); Italy). Three consecutive tests were used, the first one not considered sufficient as available data. They performed a 30 s test with eyes open followed by 30 s eyes closed.

The stand and reach test was used in order to evaluate the flexibility of the kinetic posterior chain of the legs and trunk. The participants performed a reach test 2 times on a graduated scale (Baseline, Fabrication Enterprises Retail Sales Corp, USA) and the best measure was recorded.

### Study design and statistical analysis

This was a single centre, analytic observational study, including both a cohort (PedHM, before and after exercise intervention) and a case–control (PedHM vs healthy CTRL) design. Values were expressed as mean (± SD). Sample size calculation determined that a sample of 15 participants would be adequate to detect a difference of 25% of exercise capacity (6MWT or TUDS performance) between the participants and opposed to CTRL, with a power of 0.80 (α = 0.05). D’Agostino and Pearson’s omnibus normality test was used to check if the values come from a Gaussian distribution and when the variables did not pass the normality test they were treated with non-parametric test accordingly. The statistical significance of the difference between mean values, considering the different circumstances, was evaluated as follows: (1) in case of differences between PedHM at T0, T1 vs CTRL an ordinary one-way ANOVA, followed by a Kruskall Wallis’s test, with a Dunn’s multiple comparative test was used; (2) in case of differences between PedHM at T0 vs PedHM at T1, a paired t test, two-tailed, followed by Wilcoxon matched-pairs rank test was used; (3) in case of differences between different group of PedHM (i.e. HAd vs MAd), an unpaired t test, followed by a Mann Whitney test was used. Regression analyses were performed using the least squared residuals method. The level of significance was set at *p* < 0.05. All statistical analyses were performed using a commercially-available software package (Prism 8.0: GraphPad, La Jolla, CA, USA).
